# White Tea Consumption Alleviates Anthropometric and Metabolic Parameters in Obese Patients

**DOI:** 10.3390/medicina60101568

**Published:** 2024-09-25

**Authors:** Kerimali Akyildiz, Adnan Yilmaz, Ugur Avci, Merve Nur Toraman, Zihni Acar Yazici

**Affiliations:** 1 Department of Medical Services and Techniques, School of Vocational Healh Care Services, Recep Tayyip Erdogan University, 53100 Rize, Turkey; kerimali.akyildiz@erdogan.edu.tr; 2 Department of Biochemistry, Recep Tayyip Erdogan University, 53100 Rize, Turkey; 3 Department of Endocrinology and Metabolism, Recep Tayyip Erdogan University, 53100 Rize, Turkey; ugur.avci@erdogan.edu.tr; 4 Department of Nutrition and Diet, Recep Tayyip Erdogan University, 53100 Rize, Turkey; mervenur_toraman19@erdogan.edu.tr; 5 Department of Microbiology, Recep Tayyip Erdogan University, 53100 Rize, Turkey

**Keywords:** obesity, weight loss, inflammation, lipid profile, oxidative stress

## Abstract

*Background and Objectives:* Obesity and related disorders are an increasing global health problem. Achieving and maintaining long-term weight loss through lifestyle changes and/or pharmacological interventions have not met expectations. Dietary supplements and alternative treatments have also shown limited effectiveness in this regard. The consumption of green tea in general has been shown to benefit obese patients, with effects attributed to caffeine, catechins, polyphenols and other components. However, the potential of white tea to prevent and treat the negative effects of obesity has not been addressed so far. In this study, the effect of white tea (WT) consumption in obese individuals was anthropometrically and biochemically investigated. *Materials and Methods*: Based on anthropometric and biochemical assessments, the patients were assigned to the control, orlistat, metformin and WT groups. Patients were given a diet and exercise program and one of either orlistat, metformin or WT for 12 weeks. At the end of the 12th week, the anthropometric and biochemical measurements were reassessed. *Results:* Body weight, waist circumference and BMI parameters decreased significantly in all groups. TNF-α, IL-6, IL-1β and MMP-9 levels decreased significantly in the WT group. In addition, contrary to a significant elevation in HDL-C, the serum cholesterol, LDL-C and TG levels decreased significantly. Furthermore, leptin, ghrelin and asprosin levels decreased significantly. Serum glucose levels decreased significantly in all groups except for the control. In the WT group, while there was a significant decrease in the levels of serum PL MDA and 8-OHdG, the opposite was true for GSH. *Conclusions:* The oral consumption of WT, its availability and its potency in obesity treatment and prevention pave the way for further delineation of the mechanisms of actions of its bioactive compounds at the cellular and endocrinological levels.

## 1. Introduction

Obesity is defined as an increase in body weight above the normal level as a result of excessive fat accumulation [1]. This condition is a chronic metabolic disease that increases the risk of long-term medical complications [2]. The prevalence of obesity is an increasing global public health problem [3]. Numerous conditions such as excessive and malnutrition, lack of physical activity, hormonal factors, genetic and psychological factors, and medicine use are associated with the formation of obesity. Obesity is associated with many diseases such as cardiovascular diseases, type 2 diabetes, hypertension and dyslipidemia [4,5].

Since the mechanical load and myocardial metabolism increase in obesity, oxygen consumption also increases [6]. Therefore, there is an increase in the formation of oxygen radicals (ROS) caused by mitochondrial respiration. Oxidative stress and ROS stimulate pro-inflammatory cytokine release [7]. ROS attack macromolecules such as DNA, proteins and lipids. Macromolecular damage results in cellular damage and death [8]. The damage caused by ROS is repaired by antioxidant defense systems. It has been suggested that inflammatory markers such as ghrelin, leptin, adiponectin, interleukins, matrix metalloproteinases and tumor necrosis factor are associated with insulin resistance in obese patients [9]. These substances have effects on food intake, energy balance, insulin activity, lipid and glucose metabolism, angiogenesis and vascular structuring and blood pressure in the body [10,11].

Many drugs have been used to treat obesity. Orlistat is a drug that is widely used to treat this disease. It reversibly inhibits gastric and pancreatic lipases. The inactivation of lipases prevents the hydrolysis of triglycerides [12]. Metformin, a dimethylbiguanide, inhibits the mitochondrial complex-I, which leads to AMPK (adenosine 5-monophosphate-activated protein kinase) activation [13]. By affecting the AMPK level, it provides the translocation of the glucose transporter 4 protein independently of insulin and regulates the blood glucose level. Clinicians often prescribe it to achieve weight loss [14,15]. However, they have evinced serious adverse effects, including headache, cardiovascular diseases and depression, which restrict their use. Medicines can also be expensive for patients because they are not offered with health insurance [12,16].

In recent years, one of the fastest-growing fields in the treatment of obesity is the use of natural herbal products, and many studies have proven the use of herbal products to be effective and safe. In particular, tea is reported to have anti-obesity, anti-diabetes, anti-inflammatory and hypolipidemic actions [17,18].

Tea is produced from the leaves of the Camellia sinensis plant, which belongs to the Theaceae family, and is one of the most consumed beverages after water in many societies [19]. In general, four types of tea are produced from the tea plant (Table 1). WT differs from other teas in that only the buds and young leaves of the plant are used. WT has important benefits for human health due to its high content of catechins and derivatives, as well as other tea components [20].

The aim was to investigate the effect of white tea, which is less processed and richer in contents like antioxidants and catechins than other tea varieties, on obesity. In this context, the effect of white tea consumption on the anthropometric profiles and metabolic parameters of obese patients was investigated in our study.

## 2. Materials and Methods

### 2.1. Selection of Obese Individuals

Obese individuals who applied to the Endocrinology and Metabolism outpatient clinic of R.T.E Erdogan hospital and met the selection criteria were included in this study. Informed consent was obtained from the patients. In this study, the anthropometric information of the patients, their diseases, the drugs they used, and the laboratory findings at the time of admission were examined. Patients between the ages of 18 and 65 and with a body mass index of 30 and above (kg/m^2^) were included in this study. The exclusion criteria were smoking, physical activity disability, joint problems, malignancy, trauma and cerebrovascular history. Additionally, patients using medications that could affect anthropometric and biochemical measurements were not included in this study.

### 2.2. Creation of Groups 

**Figure 1:**
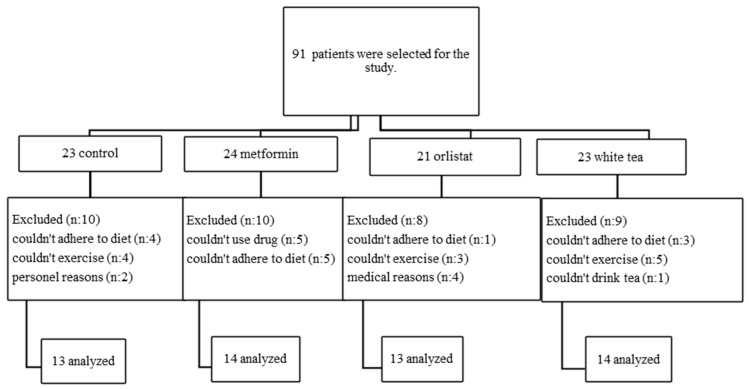
Flow chart of study set up.

Patients who met the exclusion and inclusion criteria based on anthropometric and biochemical measurements were randomly assigned into 4 groups: control (CONT, 8 females, 5 males), orlistat (ORL, 6 females, 7 males), metformin (METF, 7 females, 7 males) and white tea (WT, 6 females, 8 males). Obese patients with impaired glucose tolerance were given a diet and exercise program and METF. Those with normal glucose tolerance were given a diet and exercise program and ORL. Those who did not prefer medical treatment were given a diet and exercise program and WT. Those who did not prefer medication or WT were given only a diet and exercise program. This study lasted 12 weeks (Figure 1).

### 2.3. Medication and White Tea Use 

Metformin (1000 mg) was used twice a day and orlistat (120 mg) thrice a day. WT was prepared in the form of single-use sachets and was delivered in standard tea cups. After resting the boiled water in the tea cup for 1 min, a packet of WT was added to it. After brewing for 7 min, the tea was drunk. After drinking, the dregs and residues in the cup were eaten. Two cups of tea prepared in this way were drunk in a day. The WT used in this study was obtained by the brewing method. No extraction of any form was used. The content of the white tea used in this study was determined by high-performance liquid chromatography (HPLC) (Table 2). 

### 2.4. Implementation of Diet and Exercise

The diet program was 1400 kcal/day for women and 1600 kcal/day for men. Participants were given foods with 48% carbohydrate, 33% fat and 19% protein components. They were also advised to consume vegetables and fruits daily, and legumes and fish once or twice a week. Women were restricted to 1400 K calories and men to 1600 K calories. There was no processed food consumption. The diet program did not include anything other than WT. 

Exercise was carried out 5 days a week, for 40 min and at a moderate pace (brisk walking or swimming which slightly increased heart rate and respiratory rate, but did not cause speech difficulties). The types and amounts of all foods consumed by individuals in their main and snack meals within 24 h were recorded in detail. In addition, details of the physical activity were recorded. 

### 2.5. Follow-Up of Obese Individuals

After the anthropometric measurements and routine blood tests of the patients, those who met the study criteria were referred to a dietician. The dietitian explained the diet and exercise programs in detail to all patients. Those who could not follow the diet and exercise program were not included in the study. The patients participating in the study were interviewed by phone 2 times a week. In addition, messaging was conducted every 2 days. At the end of every 4 weeks, a face-to-face interview was held with the dietician. In these interviews, detailed information was obtained about the patients’ compliance with the programs. Those who could not comply with the schedules were excluded from the study. Anthropometric and biochemical measurements were performed again after 12 weeks in accordance with the hospital routine follow-up protocol.

### 2.6. Anthropometric Measurements 

Height (cm), body weight (kg), (Tanita BC-532 digital scale, 0.1 kg precision, Hamburg, Germany) and waist circumference (WC, cm) were measured and body mass index (BMI) was calculated.

### 2.7. Biochemical Measurements 

After 10–12 h of fasting, blood samples were taken by venipuncture into vacutainers lacking anticoagulants in accordance with the guidelines. Following the completion of coagulation, blood samples were centrifuged for 15 min at 4000 rpm. After meeting the requirements of the routine examinations, the remaining serum samples were aliquoted and stored at −80 °C until further analyses were performed [21].

Serum glucose (GLU), total cholesterol (TC), low-density lipoprotein cholesterol (LDL-C), high-density lipoprotein cholesterol (HDL-C) and triglyceride (TG) were determined with an autoanalyzer (Abbott Architect C16000, Abbott Laboratories, Abbott Park, IL, USA).

Human-specific Elabscience (Houston, TX, USA) ELISA kits were used to measure leptin (Cat No: E-EL-H6017), adinopectin (Cat No: E-EL-H6122), ghrelin (Cat No: E-EL-H1919), asprosin (Cat No: E-EL-H0515), tumor necrosis factor alpha (Cat No: E-EL-H0109), interleukin-6 (Cat No: E-EL-H6156), interleukin-1β (Cat No: E-EL-H0149), matrix metallopeptidase-9 (Cat No: E-EL-H6075), pancreatic lipase (Cat No: E-EL-H2245) and 8-hydroxy-2′-deoxyguanosine (Cat No: E-EL-0028) in accordance with the instructions of the manufacturer. 

Malondialdehyde (MDA) was measured according to Draper and Hadley’s method. MDA, the end product of lipid peroxidation, reacts with thiobarbituric acid to form a pink-colored complex that gives maximum absorbance at 532 nm [22].

The Ellman method was used to measure serum glutathione (GSH) levels. The principle of this method is to spectrophotometrically measure the color of free sulfhydryl groups in serum with the Ellman reagent [23].

### 2.8. Statistical Analysis

The SSPS 18.0 statistical program was used to evaluate the data. The Kolmogorov–Smirnov test was used to determine the distribution of data. A paired-sample *t* test was used for data that did not show normal distribution. The Wilcoxon test was performed to determine the difference between the groups. Values were expressed as median (minimum–maximum). In the analysis, *p* < 0.05 was accepted as significant.

## 3. Results

### 3.1. Anthropometric Findings

Of the 91 patients who participated in this study, 54 were able to complete the study according to the schedule. The ratios of males and females in the study groups were as follows: CONT, METF, ORL and WT. There was no significant difference in gender distribution between the groups (*p* = 0.586). The age of the patients ranged from 24 to 49 years and there was no significant difference between the groups (*p* = 0.236).

According to the first and last anthropometric measurements, there was a significant decrease in the body weight, WC and BMI parameters (*p* < 0.05). The highest decrease in anthropometric measurements was seen in the WT group (Table 3).

### 3.2. Biochemical Findings 

There was a significant decrease in serum TNF-α, IL-6, IL-1β and MMP-9 levels in the WT group (*p* < 0.05). There was a decrease in the same molecules in the other groups, too, but it was not significant (*p* > 0.05, Table 4).

While serum TC, LDL-C, HDL-C and TG levels decreased in the WT group (*p* < 0.05), this was not the case in the other groups (*p* > 0.05, Table 5).

In the WT group, serum leptin, adiponectin, ghrelin and asprosin levels decreased significantly (*p* < 0.05). There was no significant difference in the other groups (*p* > 0.05, Table 6).

There was a significant decrease in serum MDA, GSH and 8-OHdG levels in the WT group. The serum PL level decreased significantly in the CONT and ORL groups. Serum glucose levels decreased significantly in all groups except for the control (*p* < 0.05). Although there was a decrease in serum GLU, PL, MDA, GSH and 8-OHdG levels in the other groups, the difference was statistically insignificant (*p* > 0.05, Table 7).

In terms of body weight, waist circumference and body mass index parameters, there was a significant difference between the control and WT, control and METF, and control and ORL groups. There was no statistically significant difference between the groups in other parameters (*p* > 0.05, Table 8).

## 4. Discussion

In addition to nutrition and lifestyle changes, various plant polyphenols are also used in the prevention of obesity. Polyphenols have many regulatory effects on adipose tissue [24]. Polyphenols reduce lipid absorption, energy intake and lipogenesis. They also increase energy expenditure and lipolysis [25]. Tea with high amounts of polyphenols has been shown to be effective in preventing obesity. Although the effect of tea species on body weight is known, their mechanism of action is not fully known [26]. Studies are needed to elucidate the effects of WT on human metabolism. In our study, the modulatory effect of the use of WT on body weight and metabolism in obesity was examined.

Polyphenols stimulate nutrient absorption by inhibiting the enzyme PL, which is effective in lipid metabolism. The outcome is a decrease in blood glucose and lipid profile [27]. While fat oxidation and energy expenditure increase, body weight decreases. In addition, tea catechins lower glucose concentrations by inhibiting carbohydrate digestive enzymes [28]. In our study, PL decreased significantly in the WT group. Although it fell in other groups, too, it was not significant. In an in vitro study investigating PL activity, WT inhibited PL activity more than green tea [29].

In a clinical trial with metformin, exercise and WT that lasted six months, body weight and BMI decreased significantly in the exercise + WT group according to both the first and last measurements. Similarly, serum glucose, TC, LDL-C, HDL-C and TG values also decreased in the same group [30]. Like ours, this study showed that WT can provide a superior efficacy compared to metformin, along with a balanced healthy diet and regular physical exercise. A study in rats investigated the comparative effect of six types of tea (green, white, yellow, oolong, black and dark). In the study, which lasted for 8 weeks, the least weight gain was seen in the WT group. Compared to the high-fat-diet group, the highest decrease in serum glucose, TC, TG and LDL-C levels and the highest increase in HDL-C levels were seen in the WT group [31].

Texeira et al. did not see a significant difference in body weight, serum TC, HDL-C, LDL-C and glucose levels between the WT + diet group and the diet group alone in their study of obese rats for 8 weeks [32]. In another experimental animal study, animals fed a high-fat diet and given WT for 12 weeks showed a significant decrease in body weight compared to those who were not given WT [33]. These studies have shown that the duration of consumption of WT may be important for the emergence of its effects.

Obesity occurs with chronic overnutrition, high-fat and -carbohydrate diets, and excessive trans fat consumption. These diets increase superoxides, oxidative phosphorylation and glyceraldehyde oxidation, causing oxidative stress and inflammation [34]. Oxidative stress and inflammation due to obesity are proportional to the increase in adipose tissue. There is an increase in the levels of inflammatory cytokines. In addition, chronic overeating can lead to elevated enzyme levels [35,36].

In our study, there was a significant decrease in the levels of TNF-α, IL-6, IL-1β and MMP-9 inflammatory parameters in the WT group contrary to the other groups. In the WT group, there was a significant decrease in MDA levels and a significant increase in GSH levels [37]. There are other studies that show the inflammatory activity of WT against oxidative stress in obesity [31,38,39].

In the literature, there is no study on the effect of white tea on 8-OHdG levels in obesity. In rats in which oxidative stress and DNA damage were induced with benzoprene, WT significantly reduced 8-OHdG levels compared to green tea [38]. Although this study is not related to obesity, it is similar to our study in terms of oxidative stress. It showed that WT heals damaged cells.

Excesses or deficiencies of hormones can cause obesity and changes in obesity hormones. Leptin, insulin, sex hormones and growth hormones affect appetite, metabolism and body fat distribution [40]. In obese people, dysregulation in the levels of these hormones promotes abnormal metabolism and the accumulation of body fat. In addition to hormones such as leptin, which reduces energy expenditure and increases appetite, there are also hormones such as ghrelin, which reduces appetite and increases energy expenditure [41]. These hormones, which have a decisive effect on energy metabolism, contribute to the formation of appetite or satiety to a certain extent. Knowing the changes in these hormone levels is necessary for the evaluation of the obese individual [42].

A significant decrease in serum leptin and adiponectin levels was observed in the group given a high-fat diet + green tea for 4 months in obese rats [43]. Adiponectin levels increased significantly in obese women who consumed green tea, while leptin levels decreased significantly [44]. In studies comparing the effects of tea types, it was shown that green tea significantly reduced leptin and adiponectin levels compared to black tea [37]. In our study, there was a statistically more significant difference in appetite hormone levels in the WT group. This may be due to the fact that WT is richer in catechins than green tea, so the appetite-related hormones of obese individuals are affected. Under the influence of these hormones, eating habits change. 

Asprosin is a new adipokine known to increase appetite and hepatic glucose release. WT is thought to reduce the level of asprosin in obese people and improve glucose intolerance by reducing appetite [45]. In our study, in contrast to the other groups, asprosin levels decreased significantly in the WT group. Our findings are the first, as there are no studies yet linking asprosin to obesity and WT. As a result, our study showed that WT may have a protective effect against obesity by reducing asprosin levels.

## 5. Conclusions

Our results demonstrated that the consumption of WT has an important role in reducing body weight and thus preventing obesity. In addition, the results indicate that WT regulates biochemical parameters such as lipid profiles, inflammatory biomarkers, hormone levels and enzyme levels positively. However, there are very few studies on WT and the effect of WT on obesity. In these studies, anthropometric and lipid profile measurements were made. Therefore, the lack of studies on the effect of WT on obese people requires further investigation of this field.

## Figures and Tables

**Table 1:** Quality HPLC analysis of white, oolong, green and black tea samples.

	White Tea	Green Tea	Oolong Tea	Black Tea
Caffeine %	5.42	3.56	2.84	2.65
Cellulose %	8.17	15.26	16.14	15.80
Polyphenol %	18.45	15.36	11.25	9.50
Potassium %	1.86	1.27	1.34	1.18
Iron mg/100 g	77.72	158.65	166	195.02
Zinc mg/100 g	49.02	16.54	16.55	18.98
Magnesium mg/100 g	2130	1063	1840	1995
Aliminum mg/100 g	217.5	1300	1639	1817.50
Copper mg/100 g	15.65	11.51	10.45	10.02

**Table 2:** Catechin and mineral HPLC analysis results of the white tea used in this study.

Component	Amount in Dry Weight
Gallic acid	0.13 (%)
Caffeine	5.62 (%)
EGC (epigallocatechin)	2.54 (%)
EC (epicatechin)	0.91 (%)
EGCG (epigallocatechin gallate)	10.39 (%)
ECG (epicatechin gallate)	3.58 (%)
Copper	0.08 (ppm)
Iron	0.12 (ppm)
Zinc	0.54 (ppm)
Sodium	2.87 (ppm)
Potassium	283.00 (ppm)
Calcium	6.12 (ppm)
Magnesium	26.88 (ppm)
Aliminum	0.98 (ppm)

**Table 3:** Comparison of anthropometric properties.

	Weight	WC	BMI
CONT pre	88.10 (78.00–100.60)	100.50 (96.0–111.50)	32.17 (30.09–38.33)
CONT post	83.90 (72.3–96.00)	96.00 (90.50–107.00)	30.47 (27.89–36.27)
*p* value	*p* = 0.001	*p* = 0.001	*p* = 0.001
METF pre	104.70 (87.40–123.10)	112.50 (99–125)	38.92 (34.14–49.17)
METF post	95.75 (80.40–114.90)	105.50 (90–117)	35.34 (31.41–46.34
*p* value	*p* = 0.001	*p* = 0.001	*p* = 0.001
ORL pre	107.10 (80.60–140.50)	115.50 (95–139)	43.40 (40.39–45,20)
ORL post	99.50 (72.60–130.40)	105.50 (85–130.50)	39.13 (31.84–47.90)
*p* value	*p* = 0.001	*p* = 0.001	*p* = 0.001
WT pre	98.95 (86.80–129.60)	112 (99.50–127)	35.75 (30.86–48.78)
WT post	83.70 (75.30–11010)	95.75 (84–108)	30.20 (26.13–41.44)
*p* value	*p* = 0.001	*p* = 0.001	*p* = 0.001

Data are presented as median (min–max). Obtained from paired-samples *t* test. *p* value < 0.05 was considered statistically significant. Abbreviations: BMI, body mass index; CONT, control; METF, metformin; ORL, orlistat; WC, waist circumference; WT, white tea.

**Table 4:** Comparison of serum inflammatory biomarker levels.

	TNF-α (pg/mL)	IL-6 (pg/mL)	IL-1β (pg/mL)	MMP-9 (ng/mL)
CONT pre	52.97 (50.24–55.49)	22.82 (21.53–24.29)	45.76 (43.36–48.42)	3.58 (3.58–3.61)
CONT post	52.34 (50.01–55.37)	22.58 (21.40–23.79)	45.55 (42.55–47.93)	3.57 (3.22–3.60)
*p* value	*p* = 0.064	*p* = 0.133	*p* = 0.092	*p* = 0.100
METF pre	65.43 (62.29–66.80)	29.89 (26.57–31.60)	60.02 (57.14–62.57)	3.70 (3.69–3.73)
METF post	63.19 (58.59–66.36)	29.51 (25.33–30.93)	58.99 (53.85–62.56)	3.69 (3.10–3.72)
*p* value	*p* = 0.056	*p* = 0.064	*p* = 0.052	*p* = 0.173
ORL pre	66.32 (63.30–70.43)	30.73 (29.67–32.53)	69.51 (67.38–72.04)	3.77 (3.75–3.79)
ORL post	65.23 (62.51–71.27)	30.58 (28.96–32.65)	69.33 (66.75–71.68)	3.64 (2.90–3.88)
*p* value	*p* = 0.075	*p* = 0.087	*p* = 0.108	*p* = 0.055
WT pre	58.26 (56.20–60.82)	25.80 (24.02–27.63)	55.73 (53.23–57.53)	3.68 (3.67–3.72)
WT post	57.31 (54.00–60.41)	22.72 (18.06–26.82)	54.71 (51.23–57.33)	2.65 (2.63–3.71)
*p* value	*p* = 0.011	*p* = 0.006	*p* = 0.026	*p* = 0.004

Data are presented as median (min–max). Obtained from paired-samples *t* test. *p* value < 0.05 was considered statistically significant. Abbreviations: CONT, control; IL, interleukin; METF, metformin; MMP, matrix metalloproteinase; ORL, orlistat; TNF-α, tumor necrosis factor alpha; WT, white tea.

**Table 5:** Comparison of serum lipid profile levels.

	TC (mg/dL)	LDL-C (mg/dL)	HDL-C (mg/dL)	TG (mg/dL)
CONT pre	255 (233–274)	162 (155–171)	51 (46–54)	112 (100–128)
CONT post	254 (229–269)	157 (143–169)	51 (47–55)	109 (88–126)
*p* value	*p* = 0.111	*p* = 0.062	*p* = 0.110	*p* = 0.091
METF pre	267 (250–276)	147 (133–156)	50 (41–54)	110 (96–127)
METF post	265 (242–274)	139 (128–165)	52 (46–54)	103 (94–120)
*p* value	*p* = 0.080	*p* = 0.065	*p* = 0.090	*p* = 0.058
ORL pre	268 (255–279)	162 (148–188)	46 (40–55)	127 (116–148)
ORL post	266 (248–275)	159 (146–185)	48 (41–56)	122 (110–140)
*p* value	*p* = 0.054	*p* = 0.071	*p* = 0.081	*p* = 0.059
WT pre	275 (270–298)	169 (158–180)	48 (47–54)	123 (108–138)
WT post	273 (249–285)	161 (150–175)	52 (46–58)	120 (103–134)
*p* value	*p* = 0.023	*p* = 0.012	*p* = 0.007	*p* = 0.003

Data are presented as median (min–max). Obtained from paired-samples *t* test. *p*-value < 0.05 was considered statistically significant. Abbreviations: CONT, control; HDL-C, high-density lipoprotein cholesterol; LDL-C, low-density lipoprotein cholesterol; METF, metformin; ORL, orlistat; TC, total cholesterol; TG, triglyceride; WT, white tea.

**Table 6:** Comparison of serum hormone levels.

	LEP (pg/mL)	ADP (ng/mL)	GHR (ng/mL)	ASPN (ng/mL)
CONT pre	71.59 (69.76–73.98)	1.74 (1.72–1.77)	5.76 (5.70–5.79)	8.20 (8.06–8.49)
CONT post	71.51 (69.35–73.14)	1.75 (1.74–1.79)	5.74 (5.68–5.75)	8.17 (8.06–8.43)
*p* value	*p* = 0.101	*p* = 0.081	*p* = 0.091	*p* = 0.064
METF pre	72.85 (70.32–74.70)	1.72 (1.69–1.75)	5.61 (5.55–5.84)	7.99 (7.94–8.05)
METF post	72.01 (69.43–74.70)	1.74 (1.69–1.78)	5.60 (5.53–5.70)	7.93 (7.72–8.12)
*p* value	*p* = 0.066	*p* = 0.055	*p* = 0.074	*p* = 0.056
ORL pre	73.46 (70.81–75.55)	1.72 (1.70–1.74)	5.82 (5.74–5.85)	8.08 (8–8.13)
ORL post	71.70 (69.47–76.72)	1.71 (1.66–1.77)	5.79 (5.71–5.85)	8.05 (7.86–8.13)
*p* value	*p* = 0.055	*p* = 0.064	*p* = 0.075	*p* = 0.100
WT pre	72.31 (68.55–76.44)	1.74 (1.72–1.75)	5.82 (5.75–5.90)	7.88 (7.83–7.96)
WT post	70.01 (64.69–76.43)	1.75 (1.70–1.77)	5.79 (5.72–5.91)	7.82 (7.61–8.60)
*p* value	*p* = 0.019	*p* = 0.023	*p* = 0.026	*p* = 0.048

Data are presented as median (min–max). Obtained from paired-samples *t* test. *p* value < 0.05 was considered statistically significant. Abbreviations: CONT, control; ADP, adiponectin; ASPN, asprosin; GHR, ghrelin; LEP, leptin; METF, metformin; ORL, orlistat; WT, white tea.

**Table 7:** Comparison of serum GLU, PL, MDA, GSH and 8-OHdG levels.

	GLU (mg/dL)	PL (pg/mL)	MDA (nmol/g Tissue)	GSH (nmol/g Tissue)	8-OHdG (ng/mL)
CONT pre	83.0 (80.0–92.0)	65.80 (61.93–67.94)	12.13 (9.04–14.56)	15.73 (14.19–19.81)	3.83 (3.03–4.36)
CONT post	82.0 (80.0–85.0)	64.91 (62.57–66.34)	10.63 (8.63–12.11)	16.88 (14.68–23.36	3.43 (2.84–4.12)
*p* value	*p* = 0.064	*p* = 0.075	*p* = 0.055	*p* = 0.101	*p* = 0.087
METF pre	99.50 (86–106)	78.57 (76.47–81.81)	10.91 (9.61–13.09)	11.39 (9.37–13.70)	4.89 (4.39–5.34)
METF post	91.00 (81–99)	77.74 (73.64–83.42)	10.12 (6.89–13.30)	13.50 (8.31–17.85)	4.58 (4.05–5.37)
*p* value	*p* = 0.001	*p* = 0.074	*p* = 0.069	*p* = 0.056	*p* = 0.109
ORL pre	92 (83–96)	75.04 (73.09–76.98)	12.48 (11.66–14.25)	12.12 (9.85–14.71)	5.36 (4.84–5.56)
ORL post	83 (80–92)	74.28 (69.87–77.61)	11.67 (9.32–15.38)	14.27 (9.06–17.14)	5.07 (4.43–5.88)
*p* value	*p* = 0.008	*p* = 0.016	*p* = 0.064	*p* = 0.055	*p* = 0.075
WT pre	89 (81–93)	70.82 (67.70–74.52)	10.42 (9.04–12.13)	19.37 (16.30–20.59)	4.68 (4.38–5.47)
WT post	85 (80–91)	69.91 (66.17–74.24)	8.65 (5.37–12.16)	19.67 (14.46–22.12)	4.29 (3.87–5.32)
*p* value	*p* = 0.013	*p* = 0.035	*p* = 0.016	*p* = 0.022	*p* = 0.030

Data are presented as median (min–max). Obtained from paired-samples *t* test. *p* value < 0.05 was considered statistically significant. Abbreviation: CONT, control; GLU, glucose; GSH, glutathione; MDA, malondialdehyde; METF, metformin; ORL, orlistat; PL, pancreatic lipase; WT, white tea, 8-OHdG, 8-hydroxy-2′-deoxyguanosine.

**Table 8:** Results of anthropometric and biochemical analyses in all groups.

	CONT	METF	ORL	WT	*p* Value
Weight (kg)	−5.3 (−6.7/−4.2)	−7.95 (−12.60/−6.90)	−9.1 (−13/−7.4)	−14.65 (−24/−10.30)	0.001
WC (cm)	−5 (−7/−3)	−7.75 (−25/2)	−10 (−15/−8)	−15.25 (−22/−11.50)	0.001
BMI (kg/m^2^)	−1.97 (−2.49/1.34)	−2.82 (−4.63/−2.62)	−3.43 (−5.7/−2.83)	−5.37 (8.82/−3.95)	0.001

Data are presented as median (min–max). According to the results of the Kruskal–Wallis analysis, the *p* values of all groups are presented. *p* value < 0.05 was considered statistically significant (BMI, body mass index; CONT, control; METF, metformin; ORL, orlistat; WC, waist circumference; WT, white tea).

## Data Availability

The datasets used or analyzed during the current study are available from the corresponding author upon reasonable request.

## References

[1] Khati W.H., Al Mutery A.F., Ricken A., Akhigbe R.E. (2022). Progress in research on the reproductive function in the sand rat (Psammomys obesus): A review. General. Comp. Endocrinol..

[2] Zorena K., Duda O.J., Slezak D., Mrugacz R.M. (2020). Adipokines and Obesity. Potential Link to Metabolic Disorders and Chronic Complications. Int. J. Mol. Sci..

[3] Knez M., Pantovic A., Zekovic M., Pavlovic Z., Glibetic M., Zec M. (2020). Is There a Link between Zinc Intake and Status with Plasma Fatty Acid Profile and Desaturase Activities in Dyslipidemic Subjects?. Nutrients.

[4] Saltiel A.R., Olefsky J.M. (2017). Inflammatory mechanisms linking obesity and metabolic disease. J. Clin. Investig..

[5] Nakadete K., Kawakami K., Yamazaki N. (2023). Anti-Obesity and Anti-Inflammatory Synergistic Effects of Green Tea Catechins and Citrus _-Cryptoxanthin Ingestion in Obese Mice. Int. J. Mol. Sci..

[6] Boardman N.T., Pedersen T.M., Rossvol L., Hafstad A.D., Aasum E. (2020). Diet-induced obese mouse hearts tolerate an acute high-fatty acid exposure that also increases ischemic tolerance. Am. J. Physiol. Heart Circ. Physiol..

[7] Buyukuslu N., Yigitbasi T. (2015). Reactive Oxygen Species and Oxidative Stress in Obesity. J. Marmara Univ. Inst. Health Sci..

[8] Kajarabile N., Dada G. (2019). Programmed Cell-Death by Ferroptosis: Antioxidants as Mitigators. Int. J. Mol. Sci..

[9] Duraiyarasan S., Adefuye M., Manjunatha N., Ganduri V., Rajasekaran K. (2013). Colon Cancer and Obesity: A Narrative Review. Cureus.

[10] Raynor H.A., Vadivelo M. (2018). Understanding the Relationship Between Food Variety, Food Intake, and Energy Balance. Curr. Obes. Rep..

[11] Jovanovic G.K., Sutic I.M., Zezelj S.P., Susa B., Rahelic D., Majanovic S.K. (2020). The Efficacy of an Energy-Restricted Anti-Inflammatory Diet for the Management of Obesity in Younger Adults. Nutrients.

[12] Heck A.M., Yanovski J.A., Calis K.A. (2000). Orlistat, a New Lipase Inhibitor for the Management of Obesity. Pharmacotherapy.

[13] Triggle C.R., Mohammed I., Bshesh K., Marei I., Ye K., Ding H., Mcdonald R., Hollenberg M., Hill M. (2020). Metformin: Is it a drug for all reasons and diseases?. Metabolism.

[14] Doganay S., Budak O., Bahtiyar N., Toprak N. (2022). Protective effects of metformin against hepatorenal injury in high-fat diet/streptozotocine-induced diabetic mice. Kocatepe Med. J..

[15] Sari I.K., Ceylan S. (2022). The comparison of treatment with orlistat and orlistat plus metformin in relation to insulin resistance and weight loss. J. Health Sci. Med..

[16] Cheung B.M., Cheung T.T., Samaranayake N.R. (2013). Safety of antiobesity drugs. Therapeutic Advances in Drug Safety. Ther. Adv. Drug Saf..

[17] Yilmaz F., Demirel G., Kumsar A. (2016). Tea, Obesity and Women. J. Contemp. Med..

[18] Eid A., Issa L., Kamal K., Hosheya O., Sara H., Alkader S.A. (2023). Comparing and contrasting diferent herbal products intended for the management of obesity approved in the Palestinian markets. BMC Complement. Med. Ther..

[19] Akbulut A., Kara Ş.M., Ozcan A. (2020). Comparison of black, green and white teas in terms of quality criteria, mineral contents, antioxidant and antimicrobial activity. Acad. J. Agric..

[20] Yilmaz A., Dizman F., Akyildiz K., Mataraci Karakas S., Mercantepe T., Uydu H.A., Tumkaya L., Ozturk K. (2024). The Hepatoprotective Effects of Camellia sinensis on Cisplatin-Induced Acute Liver Injury. Life.

[21] Yilmaz A., Tumkaya L., Akyildiz K., Kalkan Y., Bodur A.F., Sargin F. (2017). Lasting hepatotoxic effects of prenatal mobile phone exposure. J. Matern. Fetal Neonatal Med..

[22] Drapper H.H., Hadley M. (1990). Malondialdehyde determination as index of lipid peroxidation. Methods Enzymol..

[23] Ellman G.L. (1959). Tissue sulfhydryl groups. Arch. Biochem. Biophys..

[24] Barquero S.C., Raventos R.M., Domenech M., Estruch R. (2018). Relationship between Mediterranean Dietary Polyphenol Intake and Obesity. Nutrients.

[25] Chedea V.S., Palade L.M., Marin D.E., Pelmus R.S., Habeanu M., Rotar M.C. (2018). Intestinal Absorption and Antioxidant Activity of Grape Pomace Polyphenols. Nutrients.

[26] Yang C.S., Zhang J., Zhang L., Huang J., Wang Y. (2016). Mechanisms of Body Weight Reduction and Metabolic Syndrome Alleviation by Tea. Mol. Nutr. Food Res..

[27] Chatatikun M., Kwanhian W. (2020). Phenolic Profile of Nipa Palm Vinegar and Evaluation of Its Antilipidemic Activities. Evid. -Based Complement. Altern. Med..

[28] Weyer C., Pratley R., Salbe A., Bogardus C., Ravussin E., Tataranni P. (2020). Energy Expenditure, Fat Oxidation, and Body Weight Regulation: A Study of Metabolic Adaptation to Long- Term Weight Change. JCE M^2^.

[29] Gondoin A., Grussu D., Stewart D., Mc Dougal G. (2010). White and green tea polyphenols inhibit pancreatic lipase in vitro. Food Res. Int..

[30] Pour E.D., Yaghobian F., Dehghan F., Azarbayjani M.A. (2021). Forecast of ameliorating effect of dietary flavonol consumption in white tea with or without aerobic training on type 2 diabetes (T2D) in females. Clin. Nutr. ESPEN.

[31] Zhou F., Zhu M., Tang J., Yang J., Shang B., Liu C., Wang C., Liu Q., Huang J., Liu Z. (2022). Six types of tea extracts attenuated high-fat diet-induced metabolic syndrome via modulating gut microbiota in rats. Food Res. Int..

[32] Teixeira L.G., Lages P.C., Jascolka T.L., Aguilar E.C., Soares L.P., Pereira S.S. (2012). White tea (Camellia sinensis) extract reduces oxidative stress and triacylglycerols in obese mice. Ciênc. Tecnol. Aliment. Camp..

[33] Li N., Zhou X., Wang J., Chen J., Lu Y., Sun Y., Song Y., Tan X., Xie G., Chen Y. (2022). White tea alleviates non-alcoholic fatty liver disease by regulating energy expenditure and lipid metabolism. Gene.

[34] Torres I.P., Téllez V.C., Soto M.E., Ruiz M.E., Pech L.M., Lans V.G. (2021). Oxidative Stress, Plant Natural Antioxidants, and Obesity. Int. J. Mol. Sci..

[35] Phengpol N., Thongnak L., Lungkaphin A. (2023). The programming of kidney injury in offspring affected by maternal overweight and obesity: Role of lipid accumulation, inflammation, oxidative stress, and fibrosis in the kidneys of offspring. J. Physiol. Biochem..

[36] Nahra R., Wang T., Gadde K.M., Oscarsson J., Stumvoll M., Jermutus L. (2021). Effects of Cotadutide on Metabolic and Hepatic Parameters in Adults with Overweight or Obesity and Type 2 Diabetes: A 54-Week Randomized Phase 2b Study. Diabetes Care.

[37] Lai X., Wang X., Wen S., Sun L., Chen R., Zhang Z., Li Q., Cao J., Lai Z., Li Z. (2022). Six Types of Tea Reduce Acute Alcoholism in Mice by Enhancing Ethanol Metabolism, Suppressing Oxidative Stress and Inflammation. Front. Nutr..

[38] Kumar M., Sharma V.L., Shengal A., Jain M. (2012). Protective Effects of Green and White Tea Against Benzo(a)pyrene Induced Oxidative Stress and DNA Damage in Murine Model. Nutr. Cancer.

[39] Xia X., Lin Z., Shao K., Wang X., Xu J., Zhai H., Wang H., Xu Wei Zhao Y. (2021). Combination of white tea and peppermint demonstrated synergistic antibacterial and anti-inflammatory activities. J. Sci. Food Agric..

[40] Taylor E. (2021). The complex role of adipokines in obesity, inflammation, and autoimmunit. Clin. Sci..

[41] Ozturk A., Arpaci A. (2018). The interaction of obesity and ghrelin/leptin. Mustafa Kemal Univ..

[42] Ludwig D., Aronne L., Astrup A., Cabo R., Cantley L., Friedman M. (2021). The carbohydrate-insulin model: A physiological perspective on the obesity pandemic. Am. J. Clin. Nutr..

[43] Shen C., Cao J., Dagda R., Chanjaplammootil S., Lu C., Chyu M. (2012). Green tea polyphenols benefits body composition and improves bone quality in long-term high-fat diet–induced obese rats. Nutr. Res..

[44] Huang L., Liu C., Wang L., Huang C., Hsu C. (2018). Effects of green tea extract on overweight and obese women with high levels of low density-lipoprotein-cholesterol (LDL-C): A randomised, double-blind, and cross-over placebo-controlled clinical trial. BMC Complement. Altern. Med..

[45] Zhou J., Xu C., Zhao W., Yin S., Wang G. (2022). Asprosin inhibits macrophage lipid accumulation and reduces atherosclerotic burden by up-regulating ABCA1 and ABCG1 expression via the p38/Elk-1 pathway. J. Transl. Med..

